# Massive Splenomegaly Secondary to Hematologic Dyscrasia

**DOI:** 10.7759/cureus.109074

**Published:** 2026-05-18

**Authors:** Javier Del Bosque, Daniela Salomon, Raul Verano, Diego D De La Peña, Ricardo Verano

**Affiliations:** 1 Plastic and Reconstructive Surgery, University Hospital “Dr. José Eleuterio González”, Universidad Autónoma de Nuevo León, Monterrey, MEX; 2 General Surgery, Instituto Mexicano del Seguro Social, Saltillo, MEX; 3 Surgical Oncology, Hospital Regional de Alta Especialidad del Instituto de Seguridad y Servicios Sociales de los Trabajadores del Estado, Torreón, MEX; 4 Endoscopy, Instituto Nacional de Cancerología, Mexico City, MEX

**Keywords:** choledocholithiasis, hematologic dyscrasia, hemolytic anemia, massive splenomegaly, splenectomy

## Abstract

Massive splenomegaly refers to a marked and clinically significant enlargement of the spleen beyond normal limits. Although uncommon, it is clinically significant because it may be associated with hematologic, infectious, infiltrative, malignant, or portal hypertensive disorders. Chronic hemolytic anemias are a recognized cause of marked splenic enlargement due to persistent erythrocyte destruction and reticuloendothelial hyperplasia. Ongoing hemolysis also increases bilirubin production, predisposing patients to pigment gallstone formation and biliary tract disease. We report the case of a 29-year-old male who presented with jaundice and imaging findings consistent with choledocholithiasis. His medical history was notable for chronic hemolytic anemia and recurrent episodes of jaundice. Magnetic resonance cholangiopancreatography confirmed common bile duct stones and marked splenomegaly. The patient underwent exploratory laparotomy with splenectomy and common bile duct exploration followed by choledochoduodenal anastomosis. Intraoperatively, a massively enlarged spleen and hepatomegaly were identified. Significant bleeding from the splenic pedicle required vascular control and a blood transfusion. The postoperative course was favorable, with progressive reduction in bilirubin levels and no evidence of further complications. Histopathological examination demonstrated a congestive spleen with extensive hemorrhagic areas. This case highlights the importance of a multidisciplinary approach in the evaluation and management of massive splenomegaly and illustrates that timely surgical treatment can achieve satisfactory outcomes in selected patients.

## Introduction

Massive splenomegaly is defined by the European Association for Endoscopic Surgery as a spleen measuring more than 20 cm in craniocaudal length or weighing more than 1000 g [1]. Although splenomegaly is relatively uncommon, its clinical relevance lies in the broad spectrum of underlying etiologies. Massive splenomegaly may result from several major pathophysiologic categories, including hematologic disorders, portal hypertensive conditions, infiltrative diseases, infections, and malignancies [2]. Hematologic causes include chronic hemolytic anemias, leukemias, and lymphoproliferative disorders, whereas portal hypertensive etiologies are typically associated with chronic liver disease. Infiltrative disorders such as storage diseases and systemic inflammatory conditions should also be considered in the differential diagnosis. In the present case, the clinical presentation and laboratory findings were most consistent with a hematologic etiology associated with chronic hemolysis [2].

Massive splenomegaly may result from several distinct pathophysiological processes, including hematologic disorders, portal hypertensive conditions, infiltrative diseases, and hematologic malignancies. In patients with chronic hemolytic disorders, persistent erythrocyte destruction can lead to compensatory splenic enlargement and increased bilirubin turnover, predisposing to pigment gallstone formation and recurrent biliary pathology.

Chronic hemolytic anemias are a frequent cause of significant splenic enlargement. Persistent erythrocyte destruction leads to compensatory hyperplasia of the reticuloendothelial system [3]. Additionally, ongoing hemolysis increases bilirubin production, which may precipitate as calcium bilirubinate in bile and contribute to pigment gallstone formation [4]. This association explains why patients with chronic hemolysis may develop recurrent biliary pathology even after cholecystectomy.

Persistent or recurrent common bile duct stones after cholecystectomy remain a significant clinical problem and may result in obstructive jaundice, cholangitis, recurrent biliary symptoms, and progressive biliary tract dilation. Although endoscopic management is considered first-line therapy in many patients, surgical biliary reconstruction may be necessary in complex cases involving difficult anatomy, recurrent obstruction, dense adhesions, or failed prior interventions. Choledochoduodenostomy is a well-established biliary drainage procedure that allows direct decompression of the biliary system into the duodenum and may be considered in selected patients with complex biliary disease.

This case is clinically relevant due to the coexistence of massive splenomegaly, chronic hemolytic anemia, and persistent choledocholithiasis despite prior cholecystectomy and failed endoscopic management. The case was further complicated by intraoperative hemorrhage, friable tissues, and a serosal duodenal injury requiring biliary reconstruction.

This report aims to highlight the surgical challenges and intraoperative decision-making involved in managing massive splenomegaly with concomitant biliary disease, particularly in a secondary-level hospital setting in Mexico with limited access to advanced minimally invasive resources.

## Case presentation

A 29-year-old male presented with jaundice and imaging findings suggestive of choledocholithiasis. He had a three-year history of recurrent jaundice secondary to chronic hemolytic anemia and had previously undergone laparoscopic cholecystectomy in May 2023.

Laboratory evaluation revealed anemia, with hemoglobin levels ranging from 10.2 to 12.4 g/dL. Total bilirubin levels were markedly elevated, peaking at 37 mg/dL, with both indirect (up to 12.9 mg/dL) and direct fractions (up to 29 mg/dL) elevated, consistent with a mixed pattern of hemolysis and obstructive cholestasis. Lactate dehydrogenase levels were mildly elevated (200-231 U/L). Temporal trends in liver function tests further supported the coexistence of hemolysis and biliary obstruction. AST and ALT levels demonstrated transient postoperative elevation, while alkaline phosphatase levels progressively increased from 422 U/L to 1391 U/L, consistent with worsening cholestatic obstruction. Following surgery, total and direct bilirubin levels progressively decreased, supporting successful biliary decompression.

Complete hematologic characterization was limited by the case's retrospective nature and incomplete prior records. Additional studies, including reticulocyte count, haptoglobin levels, peripheral smear analysis, direct antiglobulin testing, and evaluation for enzyme deficiency disorders, were unavailable at the time of review. Nevertheless, the combination of chronic anemia, indirect hyperbilirubinemia, pigmentary biliary disease, and massive splenomegaly was considered clinically suggestive of an underlying chronic hemolytic process.

Contrast-enhanced abdominal CT demonstrated marked splenomegaly measuring approximately 22 cm in craniocaudal length and 15 cm in transverse diameter, with displacement of adjacent structures and no focal lesions (Figures 1-2).

**Figure 1:**
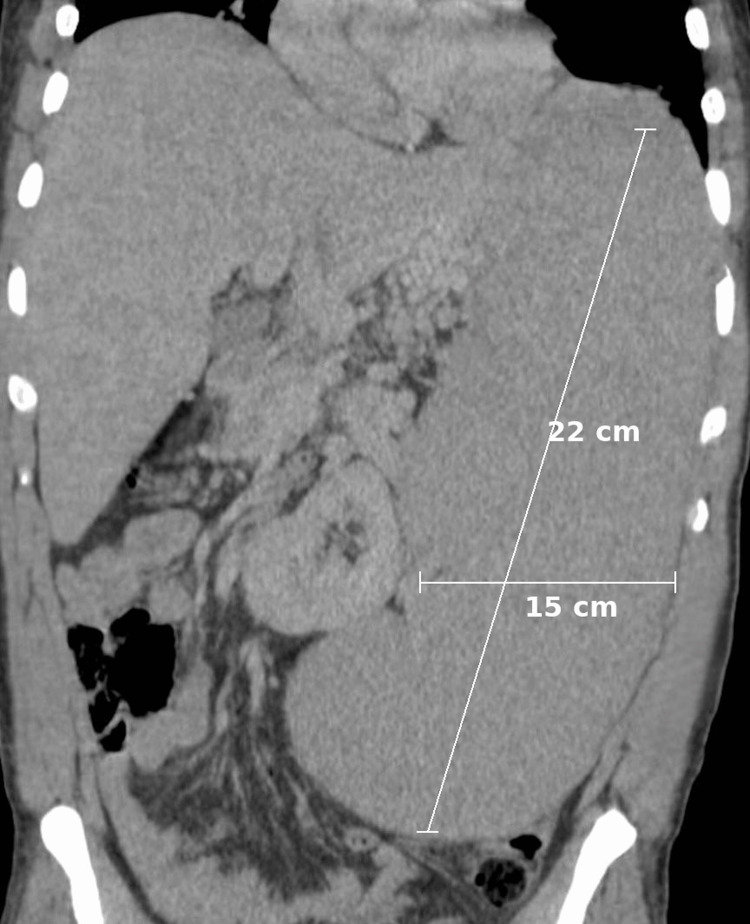
Coronal contrast-enhanced CT scan of the abdomen The coronal reconstruction demonstrates marked splenomegaly measuring approximately 22 cm in craniocaudal length, extending into the left upper quadrant with displacement of adjacent abdominal structures. CT: computed tomography

**Figure 2:**
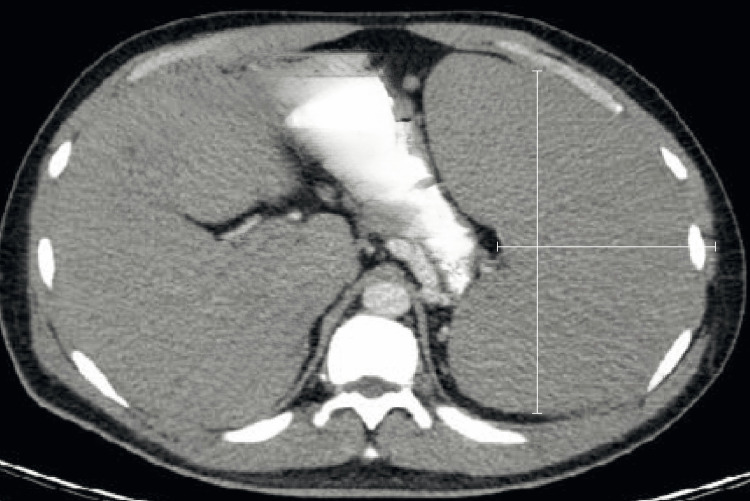
Axial contrast-enhanced CT scan of the abdomen The axial image demonstrates diffuse splenic enlargement with a transverse diameter of approximately 15 cm and homogeneous parenchymal appearance. CT: computed tomography

The patient underwent an exploratory laparotomy. A massively enlarged spleen and congestive hepatomegaly were identified. During the dissection of the splenic vascular pedicle, significant hemorrhage occurred following ligation, likely due to vessel fragility and increased vascularity associated with massive splenomegaly. This was anticipated given the splenic size and underlying hematologic condition. Hemorrhage control was achieved through vascular clamping and additional ligatures. The patient developed intraoperative hemodynamic instability requiring transfusion of three units of packed red blood cells and three units of fresh frozen plasma. Estimated blood loss was approximately 3000 mL, with part of this volume corresponding to blood contained within the enlarged spleen (Figure 3).

**Figure 3:**
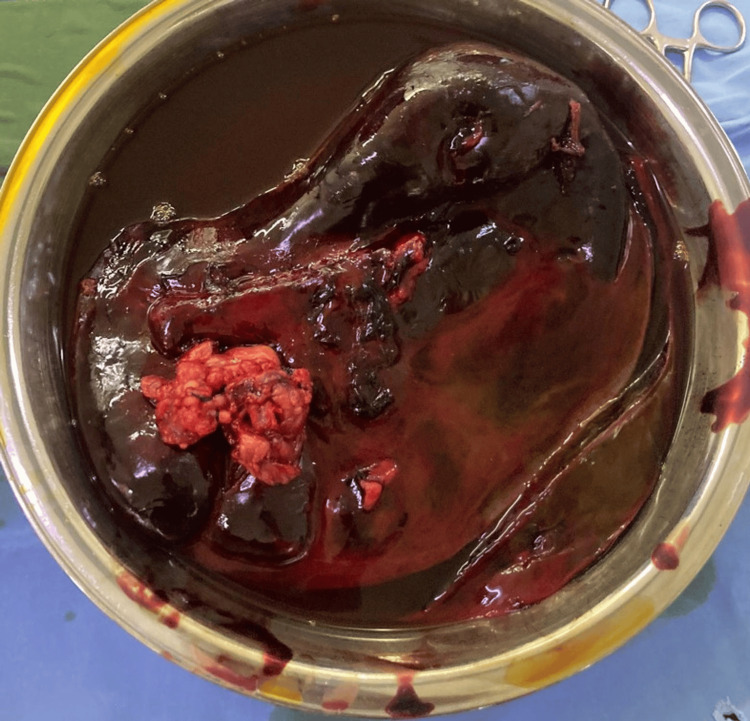
Surgically resected spleen The image shows a surgically resected spleen placed in a surgical basin immediately after splenectomy. The organ is markedly enlarged, consistent with the splenomegaly previously demonstrated on CT imaging. The spleen has a smooth but markedly distended capsule with a dark red to violaceous coloration, suggesting congestion of the splenic parenchyma. The organ appears bulky and elongated, consistent with the radiologically identified enlargement. Near the splenic hilum, there is a lobulated yellowish adipose tissue mass, likely representing attached hilar fat and vascular pedicle tissue from the splenic hilum. The surrounding surface shows areas of capsular congestion and small hemorrhagic patches. The size and congested appearance of the organ and the weight of 2,435 g. Are consistent with chronic hemolytic disease and explain the increased intraoperative bleeding risk and technical difficulty encountered during vascular pedicle dissection. CT: computed tomography

Exploration of the common bile duct revealed multiple black pigment stones, including a large stone measuring 2.7 × 2.6 cm. During dissection, a small serosal duodenal injury was identified, likely secondary to tissue friability. The lesion was recognized immediately and did not involve full-thickness perforation. Given the presence of a large obstructing stone and prior failed endoscopic management, choledochotomy with stone extraction followed by choledochoduodenal anastomosis was performed. The integrity of the repair was confirmed intraoperatively. Given the presence of a large obstructing common bile duct stone, prior failed endoscopic management, and local tissue conditions including friability, choledochoduodenostomy was selected to provide definitive biliary drainage in a single-stage procedure. Alternative options such as primary closure with T-tube drainage or hepaticojejunostomy were considered less suitable in this context.

Massive splenomegaly is associated with a substantial risk of intraoperative hemorrhage due to increased splenic vascularity, hilar distortion, and limited surgical exposure. Several strategies have been proposed to minimize blood loss, including early vascular control, vascular stapling devices, and preoperative splenic artery embolization in selected patients. In the present case, preoperative embolization was not performed because this resource was not readily available at our institution, and the patient required definitive surgical management. Despite these limitations, meticulous stepwise dissection and continuous hemostatic control allowed successful completion of the procedure.

Two 16 Fr Blake drains were placed, one directed toward the splenic fossa and the other near the biliary anastomosis for postoperative monitoring. The postoperative course was favorable, with progressive improvement in bilirubin levels and no evidence of leakage or intra-abdominal complications during follow-up.

Histopathological examination demonstrated a congestive spleen with extensive hemorrhagic areas, consistent with chronic splenic congestion in the setting of a hemolytic process. Follow-up was limited, restricting long-term assessment of complications such as portal or splenic vein thrombosis, recurrent biliary obstruction, overwhelming postsplenectomy infection, or late biliary complications.

## Discussion

Chronic hemolytic anemias such as hereditary spherocytosis and autoimmune hemolytic anemia are characterized by continuous destruction of erythrocytes, resulting in increased bilirubin turnover and splenic hyperactivity [3]. Over time, this process may produce significant splenic enlargement and predispose to the formation of pigment gallstones [4]. In the present case, chronic hemolysis likely contributed to both the massive splenomegaly and the recurrent biliary disease.

Laparoscopic splenectomy is widely considered the standard approach for normal-sized and moderately enlarged spleens because it is associated with less postoperative pain, reduced blood loss, and a shorter hospital stay compared with open surgery [1,5]. Meta-analytic data have supported these perioperative advantages in selected patients [6]. However, massive splenomegaly remains technically challenging, particularly because of limited working space, difficulty in vascular control, and increased risk of hemorrhage, which may necessitate open surgery or conversion [7]. In our patient, the size of the spleen and the coexistence of complex biliary pathology supported an open approach from the outset. Published series on massive splenomegaly have consistently demonstrated increased operative complexity, greater blood loss, longer operative time, and higher conversion rates when minimally invasive approaches are attempted in patients with very large spleens. Although laparoscopic splenectomy remains the preferred approach for normal-sized or moderately enlarged spleens, open splenectomy is still frequently recommended in cases of massive splenomegaly due to limited visualization, restricted working space, hilar distortion, and increased hemorrhagic risk. Reports describing concomitant biliary pathology with massive splenomegaly are scarce; however, similar cases emphasize the importance of individualized surgical planning based on anatomical complexity, intraoperative safety, and institutional resources. In the present case, the combination of giant splenic enlargement, extensive adhesions, and biliary involvement supported proceeding with an open approach.

Another important consideration after splenectomy is the risk of portal or splenic vein thrombosis, which has been reported as a relevant postoperative complication, particularly in patients with large spleens and hematologic disease [8]. Additionally, asplenic patients remain at lifelong risk for overwhelming postsplenectomy infection, making vaccination, patient counseling, and preventive strategies essential components of postoperative care [9].

When splenic and biliary diseases coexist, concomitant surgical management may be a reasonable strategy. Reports on combined laparoscopic splenectomy and cholecystectomy have shown that simultaneous procedures can be safe and effective in selected cases managed by experienced teams [10]. Although our patient required an open operation because of the magnitude of splenic enlargement and the complexity of biliary exploration, the principle of addressing both pathologies during the same surgical intervention remained appropriate and avoided the morbidity of staged operations.

This case emphasizes that massive splenomegaly should not be approached solely as an anatomic finding, but rather as a manifestation of an underlying systemic disorder. Careful preoperative evaluation, appropriate operative planning, and multidisciplinary perioperative management are essential to achieve favorable outcomes.

Early recognition of the underlying hematologic disorder and timely surgical intervention may prevent recurrent biliary complications and improve long-term outcomes in patients with massive splenomegaly secondary to chronic hemolytic anemia.

## Conclusions

This case illustrates the surgical complexity associated with massive splenomegaly complicated by persistent biliary obstruction after cholecystectomy. Marked splenic enlargement, distorted anatomy, dense adhesions, and increased hemorrhagic risk required individualized operative planning and multidisciplinary perioperative management.

In this patient, single-stage management with splenectomy and choledochoduodenostomy was successfully performed, resulting in improved bilirubin levels and no early postoperative complications. However, interpretation of long-term outcomes remains limited by the restricted follow-up period and the single-patient nature of the report. Although limited in generalizability, this case provides an illustrative example of the technical and perioperative challenges encountered in managing complex hepatobiliary disease with massive splenomegaly.
